# Medical Research in India

**Published:** 1917-04-14

**Authors:** 


The Workers' Newspaper of Administrative Medicine and Institutional Life, Administration, National Insurance and Health.

No. 1610, Vol. LXII. SATURDAY, APRIL 14, 1917. PRICE ONE PENNY

### MEDICAL RESEARCH IN INDIA.

When the history of modern medicine comes to be written one of its most dramatic and impressive chapters will be that in which is related the development and growth of our knowledge of the special diseases of tropical and sub-tropical latitudes. Beginning with the efforts of a few patient observers and enthusiastic students, most of whom were far removed from the stimulus of organised professional activities, this knowledge has in the course of a few years grown into a body of doctrine of high scientific import and large practical benediction. It boasts a considerable and ever-growing literature, and from its laboratories issue a continuous stream of contributions which are carried by special journals to all parts of the globe. Nor are the results less striking than the activities. Many of the records of work and discovery have a fascinating quality which must appeal to every intelligent man, and this even apart from their direct application to the prevention and treatment of disease. The explanation both of the interest and of the value of much of this work resides in the fact that it is largely concerned with the actual causes of disease. Vague surmises and loose speculations have been replaced by sure knowledge and demonstrated truths, and on these have been confidently rested methods of practice which are gradually coming into play on a large scale. Such methods concern the health of large populations and of generations yet unborn, and they promise to free from the scourge of destructive epidemic and endemic disease some of the fairest and most fruitful countries of the earth's surface. Thus the values are economic as well as scientific and humanitarian, and to quote in this respect but a single instance it is a mere frigid statement of fact to say that but for the modern abilities of tropical medicine the Panama Canal would not at this moment be in existence. The physician was the essential pioneer, and his success depended on knowledge unknown to a former generation and brought to light and put into harness by modern workers in tropical medicine. Triumphs of this order are impressive enough, but their significance is heightened by the reflection that we are yet only at the beginnings of a vast territory of truth where even now it is known there are abundant fields " white already to harvest."

The interest of the general position just stated has a special appeal to our readers when it is applied to the British Empire, with its numerous peoples and territories and the responsibilities which these involve. The moral claim, doubtless, ought to stand in the first place, but it is fair to emphasise also material considerations. With the commercial urgency which must needs follow a war budget of six millions a day, the utilitarian values of every part of the Empire acquire a new importance, both local and Imperial. And one of the hindrances to the full utilisation of these values is without question the existence of certain diseases which either with steady persistence or in epidemic outbreaks take a heavy toll of human life. What was achieved at Panama must be achieved elsewhere if economic activities are to have their due opportunity. This is one aspect of the recent discussion at the Society of Arts, introduced by the Director-General of the Indian Medical Service and dealing with medical research in India. The burden of all the speeches was to the effect that the opportunities in India for this order of work are practically unlimited, and that the existing workers are all too few. Though much has been done, there remain many questions which have still to be answered and which can only be answered with practical effect by scientific investigation. It is worth while noting that in many departments the kind of knowledge required seems at first sight to have little direct connection with the prevention and treatment of disease. Such a position largely arises from the methods by which many of the immediate causes of disease are communicated from person to person. When such agents as the flea, the louse, the mosquito, and the rest appear as distributing forces it becomes obviously necessary to gain a full knowledge of the habits and proceedings of these organisms, and, amongst other claims, the guilty species must be separated from the innocent. Hence, at the very beginning of a research whose ultimate aim is human protection we meet a demand for exact observations in a special department of natural history, and entomology must be enlisted in the service of preventive medicine. Other departments of science occupy a kindred position and must be fully explored if medical proceedings are to rest on an assured base. In short, it becomes evident that organisation must be applied to scientific research and that various classes of workers must co-ordinate their activities in relation to a common end.

The appeal, we feel sure, will not be made in vain if our medical schools are able to assure their students not only that India presents large opportunities, but also that good work done there will meet a due reward. There are welcome signs that the authorities recognise, at least in some measure, the necessity of releasing the scientific worker from the grip of official bonds and from the pressure of a Government hierarchy. This spirit needs encouragement, for it is only when she has freedom of method and freedom of thought that Science yokes herself with success to the service of mankind. The labourer is worthy of his hire, and he will prove it, provided artificial barriers are not opposed to his efforts.

